# Uncertainty Surrounding Projections of the Long-Term Impact of Ivermectin Treatment on Human Onchocerciasis

**DOI:** 10.1371/journal.pntd.0002169

**Published:** 2013-04-25

**Authors:** Hugo C. Turner, Thomas S. Churcher, Martin Walker, Mike Y. Osei-Atweneboana, Roger K. Prichard, María-Gloria Basáñez

**Affiliations:** 1 Department of Infectious Disease Epidemiology, School of Public Health, Faculty of Medicine, Imperial College London, Norfolk Place, London, United Kingdom; 2 Council for Scientific and Industrial Research, Water Research Institute, Department of Environmental Biology and Health, Accra, Ghana; 3 Institute of Parasitology, Centre for Host–Parasite Interactions, McGill University, Sainte Anne-de-Bellevue, Quebec, Canada; Lindsley F. Kimball Research Institute, United States of America

## Abstract

**Background:**

Recent studies in Mali, Nigeria, and Senegal have indicated that annual (or biannual) ivermectin distribution may lead to local elimination of human onchocerciasis in certain African foci. Modelling-based projections have been used to estimate the required duration of ivermectin distribution to reach elimination. A crucial assumption has been that microfilarial production by *Onchocerca volvulus* is reduced irreversibly by 30–35% with each (annual) ivermectin round. However, other modelling-based analyses suggest that ivermectin may not have such a cumulative effect. Uncertainty in this (biological) and other (programmatic) assumptions would affect projected outcomes of long-term ivermectin treatment.

**Methodology/Principal Findings:**

We modify a deterministic age- and sex-structured onchocerciasis transmission model, parameterised for savannah *O. volvulus–Simulium damnosum*, to explore the impact of assumptions regarding the effect of ivermectin on worm fertility and the patterns of treatment coverage compliance, and frequency on projections of parasitological outcomes due to long-term, mass ivermectin administration in hyperendemic areas. The projected impact of ivermectin distribution on onchocerciasis and the benefits of switching from annual to biannual distribution are strongly dependent on assumptions regarding the drug's effect on worm fertility and on treatment compliance. If ivermectin does not have a cumulative impact on microfilarial production, elimination of onchocerciasis in hyperendemic areas may not be feasible with annual ivermectin distribution.

**Conclusions/Significance:**

There is substantial (biological and programmatic) uncertainty surrounding modelling projections of onchocerciasis elimination. These uncertainties need to be acknowledged for mathematical models to inform control policy reliably. Further research is needed to elucidate the effect of ivermectin on *O. volvulus* reproductive biology and quantify the patterns of coverage and compliance in treated communities.

## Author Summary

Studies in Mali, Nigeria, and Senegal suggest that, in some settings, it is possible to eliminate onchocerciasis after 15–17 years of ivermectin distribution. Computer models have been used to estimate the required duration of ivermectin distribution to reach elimination. Some models assume that annual ivermectin treatment reduces the fertility of the causing parasite, *Onchocerca volvulus*, by 30–35% each time the drug is taken. Other analyses suggest that ivermectin may not have such an effect. We explore how assumptions regarding: a) treatment effects on microfilarial production by female worms (fertility), b) proportion of people who receive the drug (coverage), c) proportion of people who adhere to treatment (compliance), and d) whether people are treated once or twice per year (frequency) affect temporal projections of infection load and prevalence in highly endemic African savannah settings. We find that if treatment does not affect parasite fertility cumulatively, elimination of onchocerciasis in highly endemic areas of Africa may not be feasible with annual ivermectin distribution alone. If two areas have equal coverage but dissimilar compliance, they may experience very different infection load, prevalence and persistence trends. Projections such as these are crucial to help onchocerciasis control programmes to plan elimination strategies effectively.

## Introduction

Human onchocerciasis, caused by *Onchocerca volvulus* and transmitted by *Simulium* blackflies, is a parasitic disease leading to ocular (vision loss, blindness) and cutaneous (itching, dermatitis, depigmentation) pathology [1], [2], as well as to increased host mortality [3], [4], [5].

The Onchocerciasis Control Programme in West Africa (OCP) started in 1974. The programme was initially based on vector control until, in 1987, ivermectin was registered for human use against onchocerciasis. Thereupon, Merck & Co. Inc. took the unprecedented decision to donate ivermectin for as long as needed to eliminate onchocerciasis as a public health problem [6]. Mass drug administration (MDA) of ivermectin began in some OCP regions in 1988–1989, particularly in extension areas [7]. In some areas of the OCP both antivectorial and antiparasitic measures were combined, whilst in others (mainly in the western extension) ivermectin distribution alone, annually or biannually, was implemented [7], [8]. The African Programme for Onchocerciasis Control (APOC) was launched in 1995 to target the 19 onchocerciasis endemic countries in Africa not covered by the OCP [8], [9]. APOC's strategy involved the establishment of effective and sustainable, community-directed, annual mass ivermectin treatment for all those aged five years and older [10], [11]. The programme, initially conceived to end in 2007 [8], and subsequently in 2015 [12], has recently been extended until 2025 with the new goal and commitment for the elimination of onchocerciasis [13].

In addition to OCP western extension areas that were treated twice-yearly (e.g. Senegal [7]), some countries such as Ghana (in the former OCP), and Uganda (in APOC), have adopted a biannual treatment strategy in selected foci; the former because of suspected suboptimal responses to ivermectin treatment [14], and the latter because, in combination with vector control, elimination may be accelerated [15], [16].

Ivermectin is a potent microfilaricide, causing a greater than 90% reduction in skin microfilarial load within a few days, and a maximum reduction of 98–99% two months after treatment [17]. Ivermectin also has an embryostatic effect on adult female worms, temporarily blocking the release of microfilariae (mf) [18]. The efficacy of the embryostatic effect is approximately 70%, with the maximum reduction of microfilarial production reached one to two months after treatment [17]. Recuperation of adult worms' fertility occurs slowly from three to four months after treatment onwards [17], [18] but may not regain its original level up to 18 months after treatment. (The term fertility is used here to refer to worms producing live, stretched mf, by contrast with females producing oocytes or embryos, which would correspond to worm fecundity [17].)

Recent epidemiological and entomological evaluations conducted in Mali and Senegal suggest that 15–17 years of annual (or biannual) ivermectin distribution (in the absence of vector control) may be sufficient to lead to local onchocerciasis elimination in certain foci [19]. In addition, local elimination may have been achieved with 15–17 years of ivermectin distribution in 26 villages in Kaduna state, Nigeria (the first report of such evidence for the operational area of APOC) [20]. These studies have provided proof of principle that elimination with annual ivermectin distribution may be feasible in some African foci. In 2009, an international expert group convened to discuss the implications of these results [21]. Based on experiences with cessation of onchocerciasis control in West Africa and predictions from mathematical models, the group developed an operational framework for elimination and provisionally defined transmission thresholds, namely, a microfilarial prevalence below 5% in all surveyed villages (and below 1% in 90% of the villages), and a proportion of local simuliid vectors harbouring <0.5 L3 larvae per 1,000 flies [19], [21].

Mathematical models such as [22], have been used to assess the feasibility of, and predict the duration of ivermectin distribution required for elimination [23]. In these modelling projections, overall (therapeutic) treatment coverage was varied as part of the sensitivity analysis, and those not taking treatment included a (correlated but unreported) fraction of systematic non-compliers. However, the effect of systematic non-compliers (i.e. the proportion of the population aged five years and older who never take treatment) on the feasibility of elimination was not investigated independently from that of coverage. A crucial conjecture of these projections (based on analysis of a 5-year community ivermectin trial in Asubende, Ghana [24]), was that adult female worms, after temporarily ceasing microfilarial production due to the embryostatic effect of ivermectin, gradually reach a new production level which is reduced irreversibly by an average of 30–35% after each treatment round [25], effectively assuming a cumulative effect of ivermectin on female worm fertility (equivalent to an increasing proportion of worms not contributing to transmission; a sort of ‘macrofilaricidal’ effect [23], [25]). However, another modelling study, using data from a community trial with five biannual treatment rounds in Guatemala [26], did not find evidence for a cumulative effect on microfilarial production [27].

Whether or not ivermectin has a cumulative effect on female worm fertility [28], [29] will have important implications for the optimal design of MDA programmes, and given the sparse data that exist, this issue represents an area of considerable uncertainty which needs to be taken into account in modelling studies estimating the long-term impact of ivermectin treatment on parasite populations in humans and vectors.

In this paper, we modify our current onchocerciasis transmission model [30] to explore the uncertainty in modelling projections of the long-term impact of ivermectin on *O. volvulus* populations due to assumptions concerning: a) the effect of ivermectin on mf production by female worms (biological variables), and b) treatment coverage and compliance (programmatic variables). We also explore how these affect the benefit of annual vs. biannual treatment frequency.

## Methods

### Mathematical Model

We modified our sex- and age-structured deterministic onchocerciasis transmission model [30], [31], which describes the rate of change with respect to time and host age of the mean number of fertile and non-fertile female adult worms per host, the mean number of microfilariae per milligram (mg) of skin (mf/mg), and the mean number of infective (L3) larvae per fly. To obtain infection prevalence from infection intensity in humans, we assumed that the distribution of mf among hosts is negative binomial as described in [32]. A detailed description of the model equations is given in Supporting Information Text S1: Protocol S1, *Onchocerciasis Population Dynamics Model*. Parameter definitions and values can be found in Supporting Information Text S2: Supplementary Tables, Table S1.

### Ivermectin Effects

**Figure 1:**
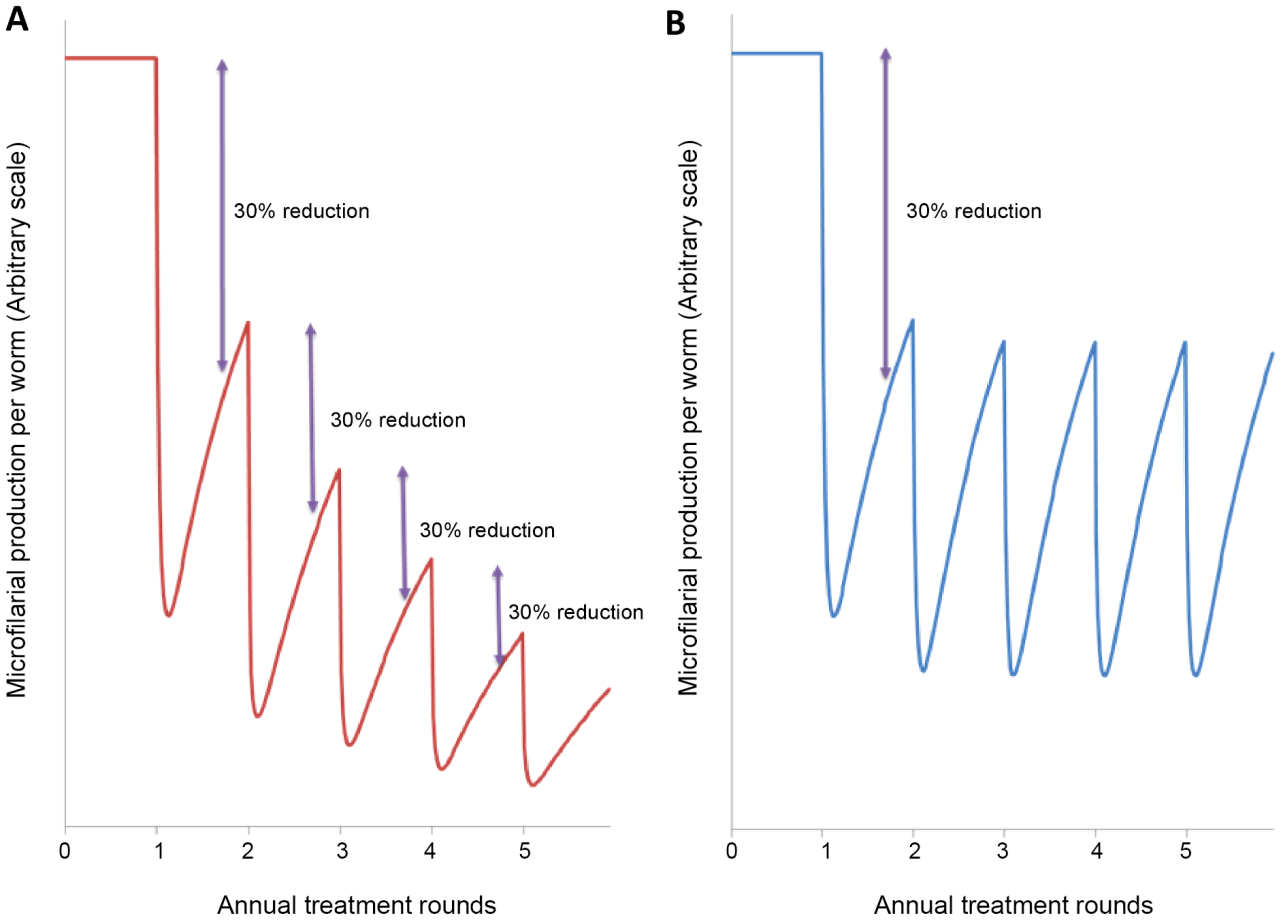
Schematic representation of two different proposed effects of ivermectin on *Onchocerca volvulus* microfilarial production. The schematic represents a closed population of adult worms (i.e., no incoming worms due to transmission or worm death). **A:** Ivermectin is assumed to have a cumulative effect on adult worm fertility by which the microfilarial production of ivermectin-exposed adult worms is reduced by 30% after each treatment round (red solid line). **B:** Ivermectin is assumed not to have a cumulative effect; ivermectin-exposed adult worms resume microfilarial production to 70% of its baseline value ten months after each treatment [17] (blue solid line).

After each dose of ivermectin there is a microfilaricidal effect with 99% efficacy, and a reduction in microfilarial production (embryostatic effect) by fertile female worms [17]. The ivermectin-exposed adult worms are then assumed either to: a) reach a new microfilarial production rate which is reduced by 30% ten months after each treatment round (representing a cumulative effect, depicted in Figure 1A), or b) resume microfilarial production, which ten months after each treatment would reach 70% of its baseline value, i.e. is also reduced by 30% from baseline, but the reduction is not additive (representing a non-cumulative effect, as concluded in [27], and illustrated in Figure 1B). The equations modelling the effect of ivermectin in female worm fertility are described in Supporting Information Text S1: Protocol S2, *Modelling the Cumulative Effect of Ivermectin*. Parameter definitions and values can be found in Supporting Information Text S2: Supplementary Tables, Table S2.

Although the cumulative reduction proposed in [25] was estimated from data corresponding to annual ivermectin distribution [24], it was assumed that in the case of biannual treatments, each 6-monthly treatment causes the same proportional reduction. An analysis of the sensitivity of model outputs to this assumption was conducted following [23]. Ivermectin was assumed to have no macrofilaricidal action (i.e. not to reduce adult worm life-expectancy) at the standard dose used for MDA [17], [33], [34], and to have intact efficacy, i.e., no sub-optimal response [14] or drug resistance [35] were included.

### Treatment Coverage, Compliance, and Frequency

The model is stratified into four treatment compliance classes: a first group of individuals who take treatment every round; two groups who take treatment every other round alternately, and a fourth group who never take treatment. The latter class represents individuals in the community who are systematic non-compliers, as opposed to a situation in which a proportion of individuals miss some treatment rounds (e.g. because they are absent or pregnant at the time of treatment). The proportion of systematic non-compliers was set at 0.1%, 2%, and 5% to investigate its effect on model outputs. These values were chosen to explore potential variability in this parameter. A recent ivermectin compliance study reported that 6% had never taken the drug over the course of eight consecutive treatment rounds [36]. The four compliance groups were assumed not to differ in exposure to vectors (which depends on age and sex according to [30]). Children under five years were not treated in the model as they are not eligible to receive ivermectin.

### Model Parameterisation and Examined Outputs

Human age- and sex-structure reflects the demography in savannah areas of northern Cameroon [37], [38], as it is in savannah areas of Africa that the prevailing *O. volvulus–S. damnosum* combinations are responsible for the most severe sequelae of onchocerciasis [1], [2]. Parameters for vector competence, survival, and host choice were those for savannah species of the *Simulium damnosum* complex (*S. damnosum sensu stricto* and *S. sirbanum*) [30], [39], responsible for onchocerciasis transmission in the region [40], [41].

The overdispersion parameter for the distribution of adult worms among hosts was as estimated in [27] (see Supporting Information Text S1: Protocol S3, *Mating Probability* and Supporting Information Text S2: Supplementary Tables, Table S3). The parameterisation of the relationship between microfilarial prevalence and load was that for West African savannah areas [32] (see Supporting Information Text S1: Protocol S4, *Microfilarial Prevalence* and Supporting Information Text S2: Supplementary Tables, Table S3). The annual biting rate (ABR) by blackfly vectors was set to 19,000 bites per person per year (well within the range of values recorded in savannah areas [32], [40], [41]), to achieve a baseline mean microfilarial load of 27 mf/mg (all ages), and of 44 mf/mg of skin in those aged 20 years and above. This resulted in an overall microfilarial prevalence (all ages) of 70%, representing an area of high baseline endemicity. In onchocerciasis, hyperendemic areas are those with overall infection prevalence above 60% [42], but this class can encompass a wide range of transmission and infection intensities. (Note that the mean microfilarial load per mg of skin in those aged ≥20 years here is an arithmetic mean, not a geometric mean of the number of microfilariae per skin snip (ss) (mf/ss) in the same age group, known as the community microfilarial load (CMFL) [43].) Understanding the long-term impact of ivermectin in highly hyperendemic areas is particularly important, as such areas will be those in which controlling the disease has the highest priority (morbidity will be more severe), elimination of the infection reservoir is likely to be more difficult or take longer [23], and from which the infection could reinvade controlled areas.

The model was used to explore the effect of 15 years of (annual or biannual) mass ivermectin distribution on: a) infection intensity defined as mean microfilarial load per mg of skin in those aged ≥20 years, and b) prevalence of microfilaridermia in the overall population. We choose 15 years as a suitable timescale to investigate the impact of long-term treatment of onchocerciasis with ivermectin, motivated by the epidemiological studies described in [19], [20]. Since the model is deterministic, the probability of reaching elimination was not investigated.

### Sensitivity Analysis

The sensitivity of the above model outputs was explored regarding the following assumptions: 1) cumulative effect of ivermectin on female worm fertility (present vs. absent); 2) overall therapeutic coverage (proportion of the total population receiving ivermectin at each round: 60%, 70%, 80%); 3) proportion of systematic non-compliers (those who never take treatment: 0.1%, 2%, 5%); and 4) treatment frequency (annual vs. biannual). In order to explore the extent to which our results were sensitive to the assumption that biannual treatments each caused the same reduction in fertility of 30% per treatment; we also explored model outputs with a more conservative reduction of 16.5% per 6-monthly treatment (which gives an overall annual reduction of 30%).

## Results

### Cumulative vs. Non-cumulative Effect of Ivermectin on Microfilarial Production by *O. volvulus*

**Figure 2:**
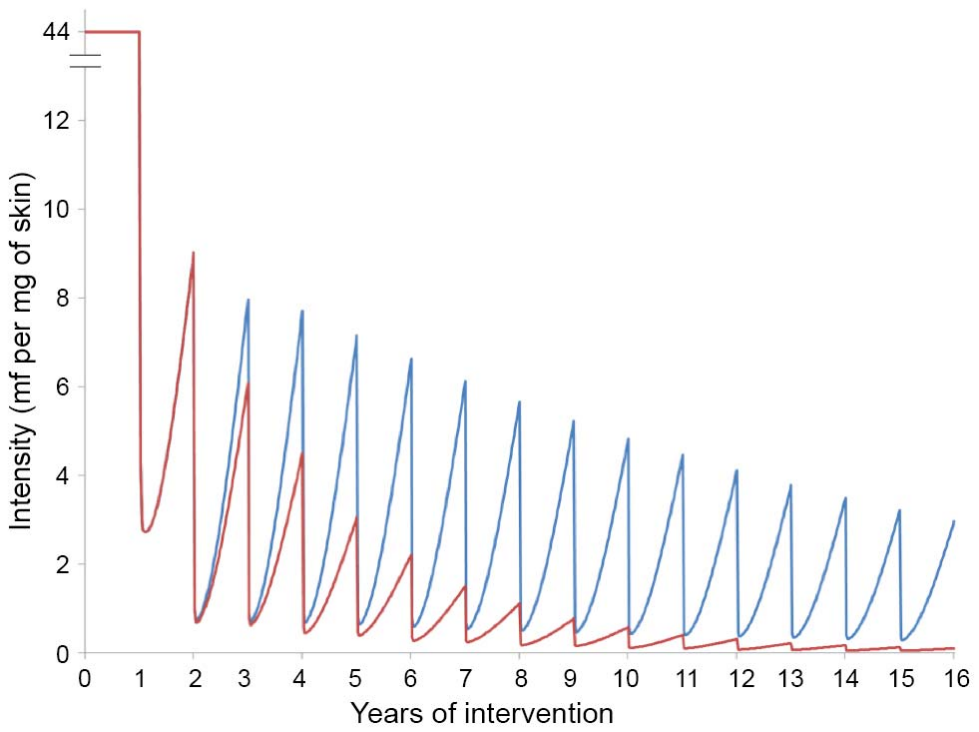
Impact on infection intensity of annual ivermectin distribution under two assumptions of ivermectin effects. Intensity of infection is quantified as microfilarial load per mg of skin in those aged ≥20 years. The red and blue solid lines represent, respectively, model outputs assuming the operation of a cumulative impact on the fertility of *O. volvulus* (illustrated in Fig. 1A), or the absence of such an effect (Fig. 1B). Model calibration corresponds to an ABR of 19,000 (savannah) *Simulium damnosum* bites/person/year; a baseline mean microfilarial load of 44 mf/mg (in those aged ≥20 years); a 70% microfilarial prevalence (all ages); a therapeutic coverage of 80% (overall population); and a systematic non-compliance rate of 0.1%. The demography of the human population is that of northern Cameroon [30], [37], [38].

Model outputs indicate that the assumption of a cumulative impact of ivermectin on microfilarial production by female *O. volvulus* has a substantial effect on projections of long-term ivermectin treatment (Figure 2). Regarding infection intensity in adults aged 20 years and older, there would be a very pronounced decrease partly due to little repopulation of the skin by mf, and partly due to the ensuing suppressed transmission. This is because, under this conjecture, the model assumes that the number of mf produced per female worm per unit time would progressively be reduced to a very low level. By contrast, under the assumption of ivermectin not exerting a cumulative effect on microfilarial production, there is a substantial amount of repopulation of the skin by mf in-between annual treatments, leading to more transmission and a smaller impact on infection intensity.

### Annual vs. Biannual Treatment Frequency

**Figure 3:**
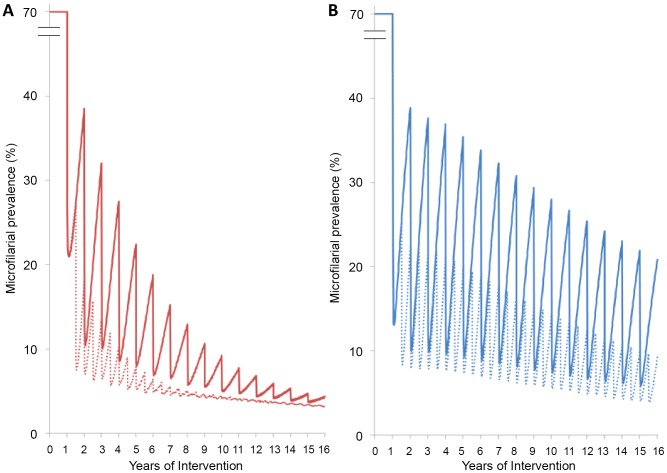
Impact on infection prevalence of annual/biannual ivermectin distribution under two assumptions of ivermectin effects. Solid and dashed lines represent, respectively, annual and biannual treatment frequency. **A:** Red lines correspond to model outputs assuming that ivermectin exerts a cumulative reduction in microfilarial production by the adult female worm. **B:** Blue lines correspond to model outputs assuming the absence of such cumulative reduction. Calibration of the model is as in Figure 2.

Assumptions regarding the operation or absence of a cumulative effect of ivermectin on parasite fertility can also influence the expected relative benefits of annual vs. biannual treatment frequency regarding reductions in infection intensity, prevalence, and transmission. In the presence of a cumulative reduction with each treatment round, there is initially a very marked benefit of the biannual distribution on the reduction of parasitological indicators (as the rate of microfilarial production is rapidly reduced). However, after repeated treatments, there would be much less difference in the long-term impact of ivermectin treatment on microfilarial prevalence compared to an annual treatment strategy (Figure 3A). In the absence of a cumulative effect, biannual treatments are more beneficial both in the short and long terms in reducing microfilarial prevalence than annual treatments (Figure 3B). With the more conservative 16.5% reduction in female fertility per 6-monthly treatment, the initial benefit of microfilarial prevalence reduction is less pronounced than in the previous scenario, but again, there is relatively little difference in the long-term impact of biannual compared to annual ivermectin treatments (Supporting Information Text S3: Supplementary Figures, Figure S1).

### Therapeutic Coverage and Compliance Patterns

**Figure 4:**
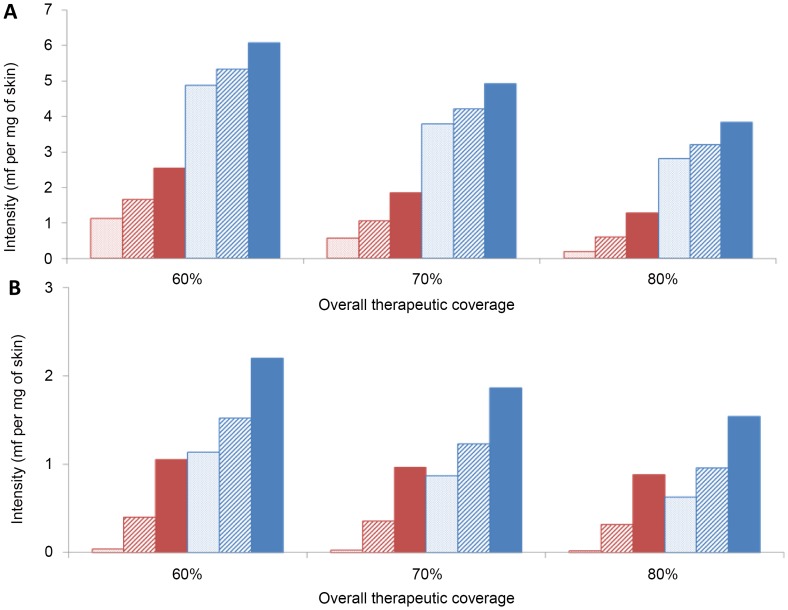
The effect of coverage and compliance on infection intensity after 15 years of ivermectin treatment. Intensity of infection is quantified as microfilarial load per mg of skin in those aged ≥20 years. The values presented correspond to one year after the 15^th^ treatment (for annual frequency, Fig. 4A), or one year after the 30^th^ treatment (for biannual frequency, Fig. 4B). Red and blue bars represent, respectively, a cumulative and a non-cumulative effect of ivermectin on microfilarial production by the female worm. Dotted bars: 0.1% systematic non-compliance; hashed bars: 2% systematic non-compliance; solid bars: 5% systematic non-compliance. Calibration of the model is as in Figure 2. Note the different scale on the vertical axis between 4A and 4B.

Varying the therapeutic coverage in the overall population, and the proportion of systematic non-compliers had a large influence on the infection intensity achieved at the end of the 15th year of ivermectin distribution. An increased overall coverage, or a decreased proportion of systematic non-compliers lead to lower microfilarial loads 12 months after the 15th year of intervention (Figure 4). Under annual treatment, overall coverage had a larger effect on projected infection intensity (Figure 4A) and microfilarial prevalence (Supporting Information Text S3: Supplementary Figures, Figure S2A) than under biannual treatment (Supporting Information Text S3: Supplementary Figures, Figure 4B and Figure S2B). (Because of the nonlinear relationship between infection prevalence and intensity, the proportional reductions in prevalence are smaller.) For instance, under the assumption of a cumulative effect of ivermectin, and for a 5% proportion of non-compliers, increasing therapeutic coverage from 60% to 80% decreased microfilarial load by ∼50% for annual frequency compared to 16% for biannual frequency. The corresponding values when no cumulative effect was assumed were ∼37% and ∼30%. By contrast, the assumed proportion of systematic non-compliers had a more pronounced effect on the impact of biannual treatment delivery. Under the assumption of a cumulative effect of ivermectin, and for a 70% therapeutic coverage, decreasing systematic non-compliance from 5% to 0.1% decreased microfilarial load by ∼69% for annual frequency and by ∼97% for biannual frequency. The corresponding values when no cumulative effect was assumed were ∼23% and ∼53%.

## Discussion

### Cumulative vs. Non-cumulative Effect of Ivermectin on Microfilarial Production by *O. volvulus*

Mathematical models can play a fundamental role in informing control programmes and strategies, but crucially, policy makers must realise that model outputs are highly dependent on implicit and explicit model assumptions [44]. Among the latter and for onchocerciasis in particular, the effects that (yearly or 6-monthly) ivermectin treatments exert on the reproductive biology of *O. volvulus* represent an area of considerable uncertainty, where further research is urgently needed. Although ivermectin's microfilaricidal effect is well established [17], the embryostatic effect and its repercussions on female worm fertility [18]; whether or not such effects on fertility are irreversible [25], [28]; the rate of resumption of microfilarial production [17]; and possible effects on intranodular sex ratios and insemination rates [45], [46], [47], remain poorly understood. An appropriate and updated incorporation of these effects into models, and an understanding of any enhanced macrofilaricidal activity of ivermectin under increased treatment frequency regimes [45], [47], [48], [49], are essential to reliably inform control policy, and fully assess ivermectin efficacy. Our results illustrate that the question of whether or not the drug effects on microfilarial production are cumulative, is highly influential on the projections of the long-term effect of annual or biannual MDA with ivermectin, particularly in areas with high baseline onchocerciasis endemicity.

The data that informed the model in [25], and presented in [24], comprised longitudinal microfilarial load follow up at various time-points after each of five annual treatment rounds in 74 individuals who received all five annual ivermectin doses from 1987 through to 1991 in an early community trial in the savannah focus of Asubende, Ghana [24]. The focus had been under vector control since 1986 during the OCP, and experienced a 70% reduction in parasite exposure during the trial despite antivectorial measures being interrupted for the first three years of ivermectin treatment. Figure 3 of [25] contrasts two model fits explaining the temporal trends in five annual data points of [24], corresponding to (decreasing) microfilarial counts just before each treatment round. The two hypotheses being tested to explain such trends are a null hypothesis of all—ivermectin-exposed—adult worms regaining their full microfilarial productivity vs. an alternative hypothesis of a 35% reduction in productivity with each treatment round. The authors of [25] concluded that the model assuming the alternative hypothesis provided a better fit to the data. However, given that: a) microfilarial loads were measured per skin snip instead of per mg of skin; b) the weight of a skin snip may range between 0.5 and 3 mg; c) lighter snips more likely yield a false negative result, and d) microfilarial counts originated from snips incubated for only 30 minutes in distilled water [24], [50] (likely to underestimate microfilarial load as microfilaridermia decreases), there is the possibility of considerable measurement error [5]. This is particularly important regarding the last two data points in the dataset (the most influential for discriminating between the two hypotheses), as for the last two years of the community trial in Asubende, the study area was receiving full vector control in addition to ivermectin, making it difficult to disentangle the effects of treatment from those of antivectorial measures. (The authors of [25] indicate, however, that the impact of vector control was taken into account in their model.) By contrast, the study in [27], based on the data presented in [26], which did not detect a cumulative effect of ivermectin on the production of microfilariae by female worms, used longitudinal data from 510 individuals (7 times as many as [24]), who took all five 6-monthly doses of ivermectin from 1998 to 1990 in the absence of vector control in Guatemala, with microfilarial loads measured per mg of skin after 24 h incubation [26].

Since our current model is deterministic, we cannot presently explore the probability of elimination. However, comparison of our projections with those of other models is informative. ONCHOSIM projections indicate that with a coverage of 80%, and an initial intensity of 70 mf/ss (in those aged 20 years and older), a minimum of 25 years of annual ivermectin distribution would be necessary to achieve a 99% probability of elimination [21]. In previous projections with the same model [23], the required duration of ivermectin distribution increases steeply and nonlinearly as heterogeneity in individual variation to vector exposure increases. Our model includes age- and sex-dependent exposure to vector bites [30] but does not consider inter-individual variation. The simulations in [21], [23] assume that ivermectin has a cumulative effect on the production of mf by female worms, and our results suggest that, in the absence of such an effect, ivermectin would have a less pronounced long-term impact. This indicates that if ivermectin does not have a cumulative effect on the fertility of *O. volvulus*, a longer duration of ivermectin distribution than previously estimated may be required to reach elimination thresholds, especially in areas with a high initial infection intensity and perennial transmission. In some areas of Cameroon that have received 13 years of ivermectin treatment, recent analyses of microfilarial dynamics do not support the operation of a strong cumulative effect of repeated treatments on the microfilarial productivity of female worms [51].

Comparison with provisional thresholds for elimination is also interesting. Operational thresholds based on [19], [21] suggest a microfilarial prevalence <5% in all of the sampled villages, or <1% in 90% of sampled villages. Our results indicate that microfilarial prevalence would remain above 5% after 15 years of annual or biannual treatment if ivermectin does not affect microfilarial production by *O. volvulus* cumulatively, even with a therapeutic coverage of 80% and only 0.1% of non-compliers (Figure 3B). Our hypothetical baseline infection levels were set at 70% microfilarial prevalence and >40 mf/mg in those aged ≥20 years, and the ABR to 19,000 bites per person per year, with perennial transmission. The baseline prevalence in the Senegalese/Malian foci reporting elimination ranged from mesoendemicity to the lower end of hyperendemicity (20% to >60%), and the CMFL from 10 to 48 mf/ss in 16 (27%) of the villages, with CMFL <10 in the remaining 44 (73%) of the 60 surveyed villages. In addition, transmission in these foci is seasonal as opposed to perennial, enhancing the impact of annual treatment on transmission when ivermectin is distributed just before the start of the rains; microfilarial loads are lowest during the transmission season and there are no blackflies around to ingest mf when these start reappearing in the skin [19]. Also, the difference with a biannual strategy would be less pronounced. These factors may have contributed to the feasibility of elimination in these areas and the reported lack of a significant difference between annual and 6-monthly treatment frequency. Likewise, in the foci located in Kaduna state, Nigeria, the median baseline prevalence was 52%, the median CMFL was 4 mf/ss, and transmission was also seasonal [20]. It should be noted that ONCHOSIM projections are consistent with current observations of elimination [19], [20], [21]. However, as described above, the areas where elimination has currently been achieved had lower baseline endemicity levels, and seasonal vector presence, leading to less transmission during inter-treatment periods. Under these conditions, assumptions of ivermectin effects on adult worms would likely have a lesser effect on models projections.

Our results are compatible with those of other modelling studies [52], which indicate that the higher the transmission intensity, the higher the necessary effectiveness of treatment (a net measure comprising coverage, number of treatment rounds per year and drug efficacy) to reach elimination. However, our study also emphasizes how different modelling assumptions can have profound effects on model outcomes and conclusions (a more extensive summary of the main structural assumptions of different onchocerciasis models is presented in [53]). This further highlights the need, discussed in [44] for helminth modellers to investigate key questions regarding helminth control more collaboratively, exploring the reasons for any disparity between the results of different models using the best available data.

### Annual vs. Biannual Treatment Frequency

Biannual ivermectin treatment was found to have a large additional benefit in both reducing microfilarial prevalence and intensity compared to annual treatment when no cumulative reduction in parasite fertility was assumed. When such effect was assumed, the model indicated that there would be an initial substantial benefit (as rates of microfilarial production are reduced quickly) of the biannual strategy, but that there would be relatively little difference in microfilarial prevalence at the end of the 15^th^ year compared to annual treatment (Figure 3A). A possible reason for the pronounced difference between the two treatment frequencies, if ivermectin does not decrease worm fertility cumulatively, is that there would be substantially more transmission between annual than between 6-monthly treatments (distributing the drug every 6 months does not allow the adult worms to regain their fertility to a substantial level if there is perennial transmission, but there may be less additional benefit in seasonal transmission scenarios). Understanding ivermectin's effect on the reproduction and survival of adult worms [17], [18], [28], [29], [45], [46], [47], [48], [49] has important policy implications regarding switching to a biannual (or more frequent) treatment strategy in Africa. Three-monthly ivermectin treatments have contributed to acceleration towards local elimination in initially hyperendemic foci in Mexico [54].

### Therapeutic Coverage

Varying therapeutic coverage (for fixed non-compliance) had less effect on the impact achieved with a biannual treatment frequency than it had for annual distribution. This can be explained as the model accounts for the fact that if someone misses a treatment round, there is another chance to get treated during that year, ensuring that at least one annual treatment is received. In annual frequency, a missed treatment would result in a gap of at least two years between treatments, allowing microfilaridermia levels to build-up and contribute to transmission in the between-treatments period. This has implications regarding policy decisions in areas that have been found to have low coverage in the past, and highlights the potential benefit of switching to a biannual treatment strategy. In any case, a higher therapeutic coverage would prevent more disease during the intervention as the intensity of infection would decrease more rapidly. Incidence of blindness [55], and relative risk of excess mortality in sighted individuals [4], [5] depend on microfilarial load. It is also important to bear in mind that our model, at this stage, does not include the possibility of sub-optimal response or resistance to ivermectin or financial costs, in which case, the described benefits of a biannual treatment frequency could be very different.

### Compliance Patterns

Assumptions regarding the proportion of systematic non-compliers were found to be just as important as those for overall coverage when projecting the long-term impact of ivermectin distribution. The proportion of systematic non-compliance (for a fixed level of therapeutic coverage) was also found to have a marked influence on the impact achieved by a biannual strategy, particularly when assuming a cumulative effect of ivermectin; the higher the non-compliance rate, the smaller the benefit of biannual treatment. This indicates that the effect of systematic non-compliance may not simply be overcome by increasing treatment frequency and has implications when considering switching to a biannual treatment strategy, as two areas with the same overall coverage but different proportion of systematic non-compliers may lead to very different results regarding the feasibility of elimination [56].

As control programmes move towards elimination goals, the proportion of systematic non-compliers in the population becomes increasingly important. Studies of coverage and compliance for lymphatic filariasis treatment have indicated that, in addition to heterogeneity in transmission and vector density, and missed rounds of MDA, continuing transmission seems to be linked to rates of systematic non-compliance [56]. Therefore, when evaluating the progress of elimination programmes, the proportion of, and factors contributing to, systematic non-compliance should be investigated in addition to those determining overall coverage [36], [57], as an assessment of the latter on its own may mask reasons behind transmission persistence.

Modelling studies should also routinely vary the proportion of systematic non-compliers in addition to levels of treatment coverage as part of their sensitivity analysis to help understand the impact of prolonged treatment in populations. Although there are some data indicating that treatment compliance may depend on host age and sex (Brieger *et al*. found that older members of the community were more likely to take ivermectin than younger sections of the population, and men were more likely to comply than women in a Cameroon, Nigeria and Uganda multi-centre study [57]), further investigation regarding patterns of systematic non-compliance (i.e. the characteristics of individuals who never take the drug) will be essential to parameterise such modelling studies.

### Conclusions and Future Directions

There is substantially more uncertainty surrounding model-derived projections of the long-term impact of, and feasibility of onchocerciasis elimination with ivermectin distribution than previously recognised. This uncertainty arises from an incomplete understanding of the effects of ivermectin on parasite survival, population structure, and reproductive biology, when the drug is administered at the standard dose annually, biannually (or more frequently, e.g. quarterly). Although the results presented in [45], [46], [47], [48], [49] would be invaluable to parameterise mathematical models incorporating such effects, further empirical and theoretical research is needed. Regarding the former, there is a need for well-characterized long-term (individual) longitudinal data (including previous treatment history), to estimate reliably the potential macrofilaricidal effects of ivermectin. However, to avoid the potentially confounding effect of ongoing transmission (which may lead to underestimating macrofilaricidal effects, particularly under annual treatment), studies could be conducted in areas where transmission has been interrupted (in geographical or ecological islands by elimination of the local vector [58], [59]). In areas near to elimination due to ivermectin distribution alone, rates of skin repopulation by mf could be investigated by fitting models to these data under a variety of ivermectin effects assumptions. Regarding the more theoretical aspects, a more adequate formulation of the parasite's mating probability in light of drug effects, decreasing male to female sex ratios [60], and changes in parasite distribution resulting from prolonged treatment [61] would also be important for assessing the feasibility of elimination.

Our results indicate that in areas with high baseline endemicity and perennial transmission, 15 years of annual or biannual treatment with ivermectin may not be sufficient to bring infection levels below potential elimination thresholds. Further incorporation of ivermectin effects into models; comparison of perennial vs. seasonal patterns of transmission; consideration of other *O. volvulus–Simulium* combinations; calibration of models for a wide range of baseline endemicity levels; assessment of patterns of treatment coverage and compliance; and inclusion of parasite genetic structure regarding sensitivity to ivermectin, will be essential to evaluate uncertainty surrounding model-derived projections. This, together with cost-effectiveness analysis, and development of stochastic frameworks will be crucial for informing control policy regarding annual vs. biannual treatment strategies in Africa, and for exploring the feasibility of elimination in foci with varying degrees of baseline endemicity. Finally, whether prolonged ivermectin treatment has a profound effect on the parasite's reproductive fitness has implications for the risk of ivermectin resistance evolving [35], and the risk of resurgence when treatment ceases. This highlights the importance of post-control surveillance in those foci where treatment is deemed to have been sufficiently successful to be stopped [62], [63], [64].

## Supporting Information

Text S1 **Model Description.**
(PDF)

Text S2 **Supplementary Tables.**
(PDF)

Text S3 **Supplementary Figures.**
(DOC)
