# Supplementary material for: Uncertainty Surrounding Projections of the Long-Term Impact of Ivermectin Treatment on Human Onchocerciasis
Source: PLoS Negl Trop Dis. 2013 Apr 25;7(4):e2169. doi: 10.1371/journal.pntd.0002169 (PMC3636241; doi:10.1371/journal.pntd.0002169)
Supplement: Text S1 — Model Description. (PDF) [file pntd.0002169.s001.pdf]

Supporting Information Text S1: Model Description

Protocol S1. Onchocerciasis Population Dynamics Model

The system of partial differential equations is based on a host age- and sex-structured onchocerciasis dynamics model presented by [1] and modified to incorporate the effects of ivermectin treatment and treatment compliance. These equations describe, respectively, the rate of change with respect to time and host age of the numbers of non-fertile, *N*, and fertile, *F*, adult female worms per host; of microfilariae per milligram of skin, *M*, and of infective (L3) larvae, *L*, per blackfly vector. The host population (and subsequently the parasite population) is partitioned into different treatment groups according to how regularly they receive ivermectin treatment (a group who takes treatment every round; two groups who take treatment every other round alternately, and a fourth group of systematic non-compliers who never takes treatment). These different compliance groups (are denoted with subscript d (their proportion in the population by η), host sex groups (males and females) with subscript s (their proportion in the population by q), τ is time since last treatment, and a is host age. Definitions and values of model parameters (for savanna *Onchocerca volvulus–Simulium damnosum s.s./S. sirbanum* in northern Cameroon [1]) are given in Table S1. Equations (omitting time and age dependencies on the left terms for simplicity, and assuming a balanced worm sex ratio) are as follows,

$$\frac{\partial N_{s,d}}{\partial t}+\frac{\partial N_{s,d}}{\partial a}=\frac{1}{2}m\beta\Omega_s(a-p)\delta_H[L(t-p)]L(t-p)\exp(-\mu_H p)+[\lambda_0+\lambda_1(\tau)]F_{s,d}(t,a)-(\varpi+\sigma_W)N_{s,d}(t,a) \qquad (S.1)$$

$$\frac{\partial F_{s,d}}{\partial t}+\frac{\partial F_{s,d}}{\partial a}=\varpi\, N_{s,d}(t,a)-[\lambda_0+\lambda_1(\tau)+\sigma_W]F_{s,d}(t,a) \qquad (S.2)$$

$$\frac{\partial M_{s,d}}{\partial t}+\frac{\partial M_{s,d}}{\partial a}=\phi[W_{s,d}(t,a),k_W]\varepsilon_d\,\psi_d(t)F_{s,d}(t,a)-[\sigma_{M_0}+\sigma_{M_1}(\tau)]M_{s,d}(t,a) \qquad (S.3)$$

$$\frac{\partial L_{s,d}}{\partial t}+\frac{\partial L_{s,d}}{\partial a}=\beta\Omega_s(a)\delta_{V_0}M_{s,d}(t,a)-\sigma_L[M_{s,d}(t,a)]L_{s,d}(t,a) \qquad (S.4)$$

$$L(t)=\sum_s\sum_d q_s\eta_d\int_a\rho(a)\Omega_s(a)L_{s,d}(t,a)\,da \qquad (S.5)$$

Intensity of infection. In the main text, intensity of infection refers to microfilarial load in those aged ≥20 years. This is calculated from Equation (*S*.3) by integrating over age (from a = 20 to $a = a_m$) and summing over sex s and compliance group d,

$$M(t)_{\geq 20} = \sum_s \sum_d q_s \eta_d \int_{a=20}^{a=a_m} \rho'(a) M_{s,d}(t,a)\, da\,, \qquad (S.6)$$

where $\rho'(a)$ is the probability density function of host age between 20 and a_m = 80 years,

$$\rho'(a) = \frac{\mu_H \exp(-\mu_H a)}{[\exp(-\mu_H 20) - \exp(\mu_H a_m)]}\,, \qquad (S.7)$$

and μ_H is the per capita death rate of humans.

Protocol S2. Modelling the Cumulative Effect of Ivermectin

At any time after the start of a simulated treatment programme, the worm population in compliance group d comprises worms previously exposed to different numbers of ivermectin treatments. This is because: a) worms continually infect hosts throughout the treatment programme, and b) hosts in different compliance groups receive different numbers of treatments at different times. If ivermectin is assumed to suppress cumulatively the fertility of female *O. volvulus*, then the average reduction in fertility of the worm population will critically depend on the fraction of worms exposed to different numbers of treatments. To this end, n was defined as the maximum number of previous exposures to ivermectin, and n + 1 sub-models were formulated to track worm populations acquired during discrete time intervals throughout the course of a simulated treatment programme. Note that n varies among compliance groups (for example, for systematic non-compliers n = 0), and that some worms, acquired after the final treatment, will be unexposed to ivermectin (j = 0). The possibility of unexposed worms gives rise to the n + 1 (as opposed to n) sub-models.

Consider a treatment programme starting at time τ' (that is, the first dose of ivermectin is administered at time $t = \tau$'). Worms exposed to all n treatments ($j = n$) are acquired at time $t < \tau$'. By redefining the rate of establishment of female adult worms from Equation (S.1) as,

$$\Lambda_s(t,a) = \frac{1}{2} m \beta \Omega_s(a-p) \delta_H [L(t-p)] L(t-p) \exp(-\mu_H p), \qquad (S.8)$$

the rate of establishment of adult worms exposed to all n treatments in compliance group d can be expressed as,

$$\Lambda_{s,d,j=n}(t,a) = \begin{cases} \Lambda_s(t,a) & \text{for} \quad 0 < t < \tau' \\ 0 & \text{otherwise.} \end{cases} \qquad (S.9)$$

By contrast, unexposed worms (j = 0) are acquired after the last treatment which, if the n treatments were administered at frequency f, indicates that infection occurred at $t > \tau' + (n-1)/f$. (In this paper we explore an annual or a 6-monthly frequency.) That is,

$$\Lambda_{s,d,j=0}(t,a) = \begin{cases} \Lambda_s(t,a) & \text{for} \quad \tau'+(n-1)/f < t < \infty \\ 0 & \text{otherwise.} \end{cases} . \qquad (S.10)$$

It follows that the rate of establishment of adult worms exposed to the intervening numbers of ivermectin treatments j = 1, 2,…, n – 1 is given by,

$$\Lambda_{s,d,j=0}(t,a) = \begin{cases} \Lambda_s(t,a) & \text{for} \quad \tau'+(n-1-j)/f < t < \tau'+(n-j)/f \\ 0 & \text{otherwise.} \end{cases} \qquad (S.11)$$

These conditions are used to define partial differential equations for the mean number of female adult worms, $W_{s,d,j}(t,a)$, in each exposure group j = 0, 1,…, n,

$$\frac{\partial W_{s,d,j}(t,a)}{\partial t}+\frac{\partial W_{s,d,j}(t,a)}{\partial a}=\Lambda_{s,d,j}(t,a)-\sigma_W W_{s,d,j}(t,a). \qquad (S.12)$$

Note that for the purposes of tracking adult worms exposed to different numbers of treatments, the fertility status (fertile/non-fertile) of female worms is not distinguished. Taking the expectation of $W_{s,d,j}(t,a)$ with respect to host age *a* and sex *s* yields,

$$W_{s,d}(t)=\sum_s q_s \int_a \rho(a)\, W_{s,d,j}(t,a)\, da \ , \qquad (S.13)$$

where $\rho(a)$, the probability density function of host age, *a*, is

$$\rho(a)=\frac{\mu_H \exp(-\mu_H\, a)}{1-\exp(-\mu_H a_m)}. \qquad (S.14)$$

Summing over exposure groups gives the mean number of worms per host in compliance group *d*,

$$W_d(t)=\sum_{j=0}^{j=n} W_{d,j}(t). \qquad (S.15)$$

The fraction of the total female worm population in ivermectin exposure group *j*, denoted $u_{d,j}(t)$, is now trivially given by,

$$u_{d,j}(t)=\frac{W_{d,j}(t)}{W_d(t)}. \qquad (S.16)$$

Each subsequent exposure to ivermectin (after the first exposure) was assumed to cause a 30% reduction in female worm fertility (see main text), such that the fertility of female worms exposed to *j* treatments, Ψ_j, is given by,

$$\Psi_j = \begin{cases} 1 & \text{for} \quad j = 0 \\ (1-\zeta)^{j-1} & \text{for} \quad j > 0 \end{cases} \qquad (S.17)$$

with parameter $\zeta = 0.3$ (Supplementary Table S2). Note that for $j = 0$ (and for $j = 1$), $\Psi_j = 1$ indicates that worms previously unexposed to ivermectin, or exposed to a single dose ($j = 1$) have, respectively, full fertility, or the potential to regain full fertility [4]. Subsequent treatments may cause a cumulative reduction of female worm fertility in this scenario.

The average reduction in female worm fertility in compliance group d, $\psi_d(t)$, is calculated using the fraction of the total worm population in each exposure group $u_{d,j}(t)$ (Equation ($S.16$)) and Ψ_j (Equation ($S.17$)),

$$\psi_d(t) = \sum_{j=0}^{j=n} \Psi_j \, u_{d,j}(t). \qquad (S.18)$$

Definitions and values of parameters are given in Supporting Table S2.

Protocol S3. Mating Probability

It is assumed that the distribution of adult worms among hosts of the same compliance group is adequately described by a negative binomial distribution (NBD) with mean (female) worm load, $W_{s,d}(t,a)$, and overdispersion parameter, k_W. Assuming polygamous mating (i.e., a single male can fertilise all females within a host) and a balanced worm sex ratio, the probability that a female worm is mated according to [2] is,

$$\phi[W_{s,d}(t,a), k_W] = 1 - \left[1 + \frac{W_{s,d}(t,a)}{k_W}\right]^{-(k_W+1)}. \qquad (S.19)$$

Note that the degree of overdispersion of the adult worm population (inversely measured by the value of k_W) is assumed to be unaffected by ivermectin treatment. Definitions and values of parameters are given in Supporting Table S3.

Protocol S4. Microfilarial Prevalence

Overall (all ages) microfilarial prevalence in compliance group d ($\pi_d(t)$) was derived by using a relationship between prevalence and microfilarial load at the community level in Cameroon described in [3]. This relationship assumes that skin microfilarial load per person is distributed according to a NBD with mean $M_d(t)$ and overdispersion parameter k_M. The best fit to the microfilarial prevalence vs. intensity relationship was obtained when k_M was allowed to be a function of the mean [3]. Assuming that the degree of microfilarial overdispersion does not depend on compliance group, $\pi_d(t)$ is given by,

$$\pi_d(t) = 1 - \left\{1 + \frac{M_d(t)}{k_M[M_d(t)]}\right\}^{-k_M[M_d(t)]} \qquad (S.20)$$

where $M_d(t)$ is given by,

$$M_d(t) = \sum_s q_s \int_a \rho(a) M_{s,d}(t,a)\, da\,, \qquad (S.21)$$

And k_M is given by [3],

$$k_M[M_d(t)] = k_0\, M_d(t)^{k_1}\,. \qquad (S.22)$$

The overall population prevalence at time t was obtained by summing $\pi_d(t)$ across compliance groups,

$$\pi(t) = \sum_{d} \eta_d \, \pi_d(t) \,. \qquad (S.23)$$

Definitions and values of parameters are given in Supporting Table S3.

References

1. Filipe JAN, Boussinesq M, Renz A, Collins RC, Vivas-Martinez S, et al. (2005) Human infection patterns and heterogeneous exposure in river blindness. *Proc Natl Acad Sci U S A* 102: 15265–15270.
2. May RM (1977) Togetherness among schistosomes: its effects on the dynamics of the infection. *Math Biosci* 35: 301–343.
3. Basáñez MG, Boussinesq M (1999) Population biology of human onchocerciasis. *Philos Trans R Soc Lond B Biol Sci* 354: 809–826.
4. Basáñez MG, Pion SDS, Boakes E, Filipe JAN, Churcher TS, et al. (2008) Effect of single-dose ivermectin on *Onchocerca volvulus*: a systematic review and meta-analysis. *Lancet Infect Dis* 8: 310–322.
