# Supplementary material for: Uncertainty Surrounding Projections of the Long-Term Impact of Ivermectin Treatment on Human Onchocerciasis
Source: PLoS Negl Trop Dis. 2013 Apr 25;7(4):e2169. doi: 10.1371/journal.pntd.0002169 (PMC3636241; doi:10.1371/journal.pntd.0002169)
Supplement: Text S2 — Supplementary Tables. (PDF) [file pntd.0002169.s002.pdf]

Turner HC, Churcher TS, Walker M, Osei-Atweneboana MY, Prichard RK, Basáñez MG. Uncertainty surrounding projections of the long-term impact of ivermectin treatment on human onchocerciasis

Supporting Information Text S2: Supplementary Tables

Table S1. Definition and values of parameters and variables for the onchocerciasis population dynamics model

Symbol	Definition of variables and parameters	Expression, average value and units	Ref.
	Pertaining to human host		
$N_{s,d}(t,a)$	Mean number of non-fertile female adult worms per person at time (*t*) and age (*a*); subscript *s* denotes host sex and *d* denotes treatment compliance category	Equation (*S*.1)	Protocol S1
$F_{s,d}(t,a)$	Mean number of fertile female adult worms per person at time (*t*) and age (*a*); subscripts *s* and *d* as above	Equation (*S*.2)	Protocol S1
$M_{s,d}(t,a)$	Mean number of microfilariae (mf) per milligram of skin at time (*t*) and age (*a*); subscripts *s* and *d* as above	Equation (*S*.3)	Protocol S1
$\delta_H[L(t)]$	Proportion of L3 larvae developing to adult worms within the human host as a function of the number of infective larvae received per unit time	$\frac{\delta_{H_0} + \delta_{H\infty} c_H m\beta L(t)}{1 + c_H m\beta L(t)}$	[1]
δ_{H_0}	Proportion of L3 larvae developing to adult worms within the human host when $m\beta L(t) \to 0$	0.0854	[2]
δ_{H_∞}	Proportion of L3 larvae developing to adult worms within the human host when $m\beta L(t) \to \infty$	0.00299	[3]
c_H	Severity of transmission intensity-dependent parasite establishment within the human host	5.86 x 10^{-3} yr per L3 larva	[2]
μ_H	Per capita death rate of human hosts	0.04 yr^{-1}	[2]
σ_W	Per capita death rate of adult worms	0.1 yr^{-1}	[1]
σ_{M_0}	Per capita death rate of microfilariae in the absence of ivermectin	0.8 yr^{-1}	[1]
ϖ	Per capita rate at which untreated, non-reproducing female worms become fertile	0.59 yr^{-1}	[4]
λ_0	Per capita rate at which untreated fertile female worms become non-fertile in the absence of ivermectin	0.33 yr^{-1}	[4]
ε_d	Rate of production of microfilariae per fertile female worm scaled by the total weight (in milligrams) of microfilariae-bearing skin	1.1538 yr^{-1}	[1]
a_m	Maximum recorded human age in the reference population of northern Cameroon	80 yr	[2]

Table S1. Continued

Symbol	Definition of variables and parameters	Expression, average value and units	Ref.
	Pertaining to human host (continued)		
p	Prepatent period (from infection with L3 larvae to presence of detectable microfilariae in the skin)	2 yr	[2]
$\rho(a)$	Probability density function of host age *a* (using a truncated exponential distribution of survival times)	$\dfrac{\mu_H \exp(-\mu_H\, a)}{1-\exp(-\mu_H a_m)}$	[2]
η_d	Proportion of the host population in compliance group *d*	–	Protocol S1
q_s	Proportion of the host population in sex group (females/males) *s*	0.45/0.55	[2]
	Pertaining to simuliid vector		
$L(t)$	Mean number of infective larvae per fly at time (*t*)	Equation (*S*.5)	Protocol S1
m	Vector to host ratio	609, for ABR = 19,000 bites person^{-1} yr^{-1}	For 70% prevalence
β	Biting rate per fly on humans assuming a human blood index = 0.3	31.2 yr^{-1}	[1]
δ_{V_0}	Proportion of ingested microfilariae developing to the infective stage within the vector, per bite	0.005	[2]
$\sigma_L[M_{s,d}(t,a)]$	Per capita net rate of loss of L3 larvae from vectors	$(a_H / g)+\sigma_{L_0}+\mu_V+\alpha_V M_{s,d}(t,a)$	[1]
a_H	Proportion of infective, L3 larvae shed per bite	0.5	[2]
g	Average duration between consecutive blood-meals	0.0096 yr	[1]
σ_{L_0}	Per capita death rate of L3 larvae within the vector	104 yr^{-1}	[1,3]
μ_V	Per capita death rate of uninfected blackflies	52 yr^{-1}	[1,3]
α_V	Parasite induced death rate of infected blackflies	0.6 yr^{-1} per microfilaria	[2]
$\Omega_s(a)$	Age- and sex-specific measure of exposure to vectors	$\begin{cases} E_s\gamma_s E_0,\ a<a' \\ E_s\gamma_s \exp[-\alpha_s(a-a')],\ a>a' \end{cases}$	[2]
E_s	Sex-specific exposure to vector bites (females/males)	0.90/1.08	[2]
E_0	Fraction of exposure at age 0 in relation to that at age *a*’ from which exposure is allowed to change with age	0.10	[2]
γ_s	Normalisation factors to ensure that the distribution of bites among age groups sums to 1 (females/males)	0.548/1.154	[2]
α_s	Age-specific change in contact rate with vectors for human hosts of sex *s* (females/males)	–0.023/0.007	[2]

Table S2. Definition and values of parameters and variables for ivermectin treatment effects

Symbol	Definition of variables and parameters	Expression, average value and units	Ref.
n	Maximum number of previous exposures to ivermectin by worms in a given compliance group	0 for those hosts never taking treatment to 15 (annual) or 30 (biannual) for those taking all treatments	Protocol S2
f	Frequency of treatment	Annual or biannual	Protocol S2
τ	Time since last ivermectin treatment	years	–
$\lambda_1(\tau)$	Excess per capita rate at which fertile females become non-fertile following ivermectin treatment (embryostatic effect)	$32.4\exp(-19.6\tau)\,\text{yr}^{-1}$	[4]
$\sigma_{M_1}(\tau)$	Excess per capita death rate of microfilariae following ivermectin treatment (microfilaricidal effect)	$\left(\tau+9.6\times10^{-3}\right)^{-1.25}\text{yr}^{-1}$	[4]
τ’	Treatment programme start time	–	–
$\Lambda_{s,d,j}(t,a)$	The rate of establishment of female adult worms at time *t* in hosts of age *a*, sex *s*, treatment compliance group *d* and exposure group (number of treatments to which worms have been exposed to) *j*	Equations (*S*.9) and (*S*.10)	Protocol S2
$W_{s,d,j}(t,a)$	Mean number of female adult worms at time (*t*) and age (*a*); *s* denotes sex, *d* denotes treatment compliance category and *j* ivermectin exposure group	Equation (*S*.12) and (*S*.13)	Protocol S2
$u_{d,j}(t)$	The fraction of the total worm population in exposure group *j*	Equation (*S*.16)	Protocol S2
Ψ_j	The net reduction in fecundity of adult worms in exposure group *j*	Equation (*S*.17)	Protocol S2
ζ	The per dose reduction in fecundity caused by ivermectin when a cumulative effect is assumed	0.30	[5]
$\psi_d(t)$	The average reduction in fertility in compliance group *d*	Equation (*S*.18)	Protocol S2

Table S3. Definition and values of parameters for mating probability and microfilarial prevalence calculations

Symbol	Definition of variables and parameters	Expression, average value and units	Ref.
$\phi[W_{s,d}(t,a),k_W]$	Mating probability at time *t*, age *a*, sex *s* and treatment compliance group *d*	Equation (*S*.19)	Protocol S3
$W_{s,d}(t,a)$	Mean number of female adult worms per person at time (*t*) and age (*a*), *s* denotes sex and *d* denotes treatment compliance category	$N_{s,d}(t,a)+F_{s,d}(t,a)$	Protocol S3
k_W	Inverse measure of degree of overdispersion in the distribution of worms among hosts	0.35	[6]
$\pi_d(t)$	Microfilarial prevalence at time *t* in compliance group *d*	Equation (*S*.20)	Protocol S4
$k_M[M_d(t)]$	Inverse measure of the degree of overdispersion in the distribution of skin microfilariae among hosts of compliance group *d*, as a function of the mean microfilarial load	Equation (*S*.22)	[1]
k_0	Parameters of the relationship between k_M and skin microfilarial load	0.0553	[1]
k_1		0.4910	[1]

References

1. Basáñez MG, Boussinesq M (1999) Population biology of human onchocerciasis. *Philos Trans R Soc Lond B Biol Sci* 354: 809–826.
2. Filipe JAN, Boussinesq M, Renz A, Collins RC, Vivas-Martinez S, et al. (2005) Human infection patterns and heterogeneous exposure in river blindness. *Proc Natl Acad Sci U S A* 102: 15265–15270.
3. Basáñez MG, Collins RC, Porter CH, Little MP, Brandling-Bennett D (2002) Transmission intensity and the patterns of *Onchocerca volvulus* infection in human communities. *Am J Trop Med Hyg* 67: 669–679.
4. Basáñez MG, Pion SDS, Boakes E, Filipe JAN, Churcher TS, et al. (2008) Effect of single-dose ivermectin on *Onchocerca volvulus*: a systematic review and meta-analysis. *Lancet Infect Dis* 8: 310–322.
5. Plaisier AP, Alley ES, Boatin BA, Van Oortmarssen GJ, Remme JH, et al. (1995) Irreversible effects of ivermectin on adult parasites in onchocerciasis patients in the Onchocerciasis Control Programme in West Africa. *J Infect Dis* 172: 204–210.
6. Bottomley C, Isham V, Collins RC, Basáñez, MG (2008) Rates of microfilarial production by *Onchocerca volvulus* are not cumulatively reduced by multiple ivermectin treatments. *Parasitology* 135: 1571–1581.
