# Supplementary material for: Uncertainty Surrounding Projections of the Long-Term Impact of Ivermectin Treatment on Human Onchocerciasis
Source: PLoS Negl Trop Dis. 2013 Apr 25;7(4):e2169. doi: 10.1371/journal.pntd.0002169 (PMC3636241; doi:10.1371/journal.pntd.0002169)
Supplement: Text S3 — Supplementary Figures. (DOC) [file pntd.0002169.s003.doc]

**Supporting Information Text S3: Supplementary Figures**

**Figure S1**

**A comparison of the long-term impact of annual and biannual treatment strategies on microfilarial prevalence.** Solid and dashed lines denote, respectively, annual and biannual ivermectin treatment frequency. Results assume that ivermectin exerts a cumulative reduction in the rate of microfilarial production by female worms, with a more pessimistic 16.5% reduction per treatment when frequency is biannual. Model calibration corresponds to an ABR of 19,000 (savannah) *Simulium damnosum* bites/person/year; a baseline mean microfilarial load of 44 mf/mg (in those aged ≥ 20 years); a 70% microfilarial prevalence (all ages); a therapeutic coverage of 80% (overall population); and a systematic non-compliance rate of 0.1%.

**Figure S2**

**The effect of coverage and compliance on microfilarial prevalence after 15 years of ivermectin treatment.** The values presented correspond to those obtained one year after the 15^th^ treatment (for annual frequency, Fig. S2A), or one year after the 30^th^ treatment (for biannual frequency, Fig. S2B). Red and blue bars represent, respectively, a cumulative and a non-cumulative effect of ivermectin on microfilarial production by the female worm. Dotted bars: 0.1% systematic non-compliance; hashed bars: 2% systematic non-compliance; solid bars: 5% systematic non-compliance. Calibration of the model is as in Figure S1.
